# Evaluating the feasibility and acceptability of home-based isometric exercise and behaviour change for the management of hypertension: The HOME-FIT study protocol

**DOI:** 10.1371/journal.pone.0353111

**Published:** 2026-07-10

**Authors:** Helen Llewellyn, Sarah Audsley, Paul Court, Angela Rodrigues, Eurico Wilhelm Neto, Gabriel Grizzo Cucato

**Affiliations:** 1 School of Sport, Exercise and Rehabilitation, Northumbria University, Newcastle upon Tyne, United Kingdom; 2 Healthworks NE, Newcastle upon Tyne, United Kingdom; 3 School of Psychology, Northumbria University, Newcastle upon Tyne, United Kingdom; PLOS: Public Library of Science, UNITED KINGDOM OF GREAT BRITAIN AND NORTHERN IRELAND

## Abstract

This protocol describes a pilot randomised controlled trial that aims to assess the feasibility and acceptability of a 24-week remotely delivered home-based isometric exercise and behaviour change intervention for adults with arterial hypertension (AH) characterised as ≥140/90 mmHg by European and UK guidelines. The study will also explore the potential effects of the intervention on office and ambulatory blood pressure (BP). Seventy participants diagnosed with AH and receiving pharmacological treatment will be recruited from Newcastle upon Tyne, United Kingdom. Participants will be randomised with minimisation for sex and mean office systolic BP (≤140 mm Hg vs > 140 mm Hg) to the intervention (n = 35) or control group (n = 35). The intervention group will undergo 12 weeks of remotely supervised isometric wall squat exercises, three times per week, and will receive advice based on a leaflet that follows UK recommendations for behaviour change, including weight management, diet, physical activity, salt intake, alcohol reduction, and smoking cessation. After that, they will be followed for an additional 12 weeks and will continue to receive further guidance to maintain the wall squat exercise and behaviour changes. The control group will receive similar advice to that of the intervention group, reinforcing the standard care recommendation. Primary outcomes for feasibility will be determined by screening, eligibility, recruitment, and retention rates at 12 and 24 weeks. Exercise adherence will be measured by the proportion of sessions completed. Behaviour change adherence will be self-reported via a questionnaire and assessed at 12 and 24 weeks. Participant acceptability will be determined through semi-structured interviews. Secondary outcomes include office and ambulatory BP measurements at baseline, 12 weeks, and 24 weeks. Findings will guide the development of a future large-scale trial to evaluate the effectiveness and cost-effectiveness of non-pharmacological hypertension management, addressing key gaps and supporting scalable, patient-centred care.

ClinicalTrials.gov Registration Number: NCT07213479

## Introduction

Arterial hypertension (AH) is a leading risk factor for cardiovascular diseases, contributing significantly to global morbidity, mortality, and economic burden [1]. Findings from the Global Burden of Disease report list AH as one of the three leading risk factors for premature death and poor health worldwide [2]. In the United Kingdom (UK), mortality from cardiovascular disease accounts for approximately 27% of all deaths in the adult population, representing more than 160,00 deaths per year, or one death every 3-minutes [3]. Moreover, cardiovascular diseases impact the UK economy, with an estimated cost of nine billion pounds per year [4].

One of the main risk factors for cardiovascular disease is AH, characterised in the UK and European Guidelines as systolic blood pressure (BP) equal to or higher than 140 mmHg and/or diastolic BP equal to or higher than 90 mmHg [5,6]. Recent data from the World Health Organisation indicated that worldwide an estimated 1.28 billion adults aged 30–79 years have AH [1]. Specifically, in the UK, approximately 30% of all adults have AH, with most not receiving effective treatment to control their BP [7]. Public Health England suggested that better detection and management of high BP could prevent more than 9,000 heart attacks and 14,000 strokes over a 3-year period [8].

Non-pharmacological strategies through lifestyle modifications have been consistently recommended as the cornerstone of therapy for the prevention and treatment of individuals with AH, as reductions in BP levels have been associated with lower cardiovascular risk and mortality [6,9]. Current guidelines for the management of AH include exercise training and lifestyle advice for modifiable risk factor changes, such as smoking cessation, reduced sodium intake and alcohol consumption [10]. Generally, exercise recommendations have focused on aerobic activities at moderate to vigorous intensity with a vast body of literature demonstrating favourable outcomes [11]. Additionally, dynamic resistance training has been recommended for individuals with AH to improve overall strength yielding similar benefits as aerobic exercise [6]. Although crucial for non-pharmacological AH management, adherence to these traditional modalities of exercise training is low due to many barriers (13), such as a lack of time (session duration of at least 30–60 minutes), costs, and accessibility (gym subscription or specialised supervision), which pose challenges for long-term adherence.

Isometric exercise, characterised by muscular contraction without movement of the surrounding joints, has emerged as an alternative method for managing and treating AH. Isometric exercise can be performed for the upper (e.g., handgrip) and lower limbs (e.g., leg extension and wall squat), offering significant advantages, including a minimal time commitment (approximately 11–20 minutes), minimal or no equipment requirements, and accessibility as instruction can be given in person or remotely. There is a growing body of research investigating the effects of isometric exercise training (IET) for BP management. A multilevel meta-review and regression analysis [12] exploring the effects of IET for pre and hypertensive individuals concluded effectiveness in reducing office BP of −7 mmHg, −4 mmHg, and – 6 mmHg for systolic, diastolic, and mean BP respectively. Furthermore, a network meta-analysis exploring the potential best exercise for BP reduction demonstrated from all modalities, leg IET, specifically the wall squat, presented the greatest reduction in office BP, with −11 and −5 mmHg for systolic and diastolic BP, respectively [13]. One possible explanation of these findings may be associated with ischemia/reperfusion muscle mass recruitment with leg IET. It may be hypothesised that large muscle mass isometric exercise (such as that involving lower limbs) may induce a more pronounced ischemia-reperfusion response due to sustained muscle contraction and higher intramuscular pressure, leading to increased sympathetic-mediated vasoconstriction during exercise and enhanced post-exercise vasodilation [14]. This process may result in a significant reduction in systemic vascular resistance. Additionally, leg IET may exert favourable effects on autonomic cardiovascular regulation, potentially enhancing parasympathetic tone and reducing sympathetic activity [15], thereby contributing to the observed reductions in BP [16].

Current evidence supports further investigation into the use of the leg IET as an alternative modality for AH. However, to advance this area of research, several important gaps in current literature must be addressed. To date, only four studies [17–20] have investigated the effects of wall squat training, primarily in normotensive, pre-hypertensive, or non-medicated stage 1 hypertensive populations as defined by NICE classification guidelines. Although these studies elicited favourable BP reductions, it remains unclear whether these findings are generalisable to individuals with established AH, who represent the population with the greatest need for effective BP management. A critical gap in research is the absence of interventions that integrate behavioural strategies aimed at promoting long-term lifestyle changes. Furthermore, prior studies have not integrated behavioural strategies to support long-term lifestyle change. Both NICE and European guidelines emphasise that individuals with AH should receive structured lifestyle advice to increase physical activity, improve diet, and reduce smoking and alcohol consumption. Such strategies not only can reduce BP but also address co-existing risk factors, including diabetes, obesity, hypercholesterolaemia, and CVD [2].

Considering these knowledge gaps, we propose a pilot study to evaluate the feasibility and acceptability of a combined intervention incorporating remotely isometric wall squat training and behavioural support advice. We will assess adherence, acceptability, and preliminary exploratory BP effects. Findings will inform the design and sample size of a fully powered, multicentre randomised controlled trial to evaluate clinical effectiveness, wider cardiometabolic outcomes, and cost-effectiveness for integration into NHS services.

## Aims of the study

### Primary aim

To determine:

- Rates of participant screening, eligibility, recruitment, and retention at 12 and 24 weeks.
- Participant adherence to intervention (number of sessions attended and completed)
- Patient acceptability of the intervention through semi-structured qualitative interviews.

### Secondary aim

To assess the feasibility and acceptability of collecting clinical and ambulatory BP data, considering the perspectives of participants and Healthcare clinical professionals, as well as data collection challenges and missing data rates. Findings will inform the suitability of these measures for a future full-scale trial.

## Materials and methods

### Study design

**Fig 1:**
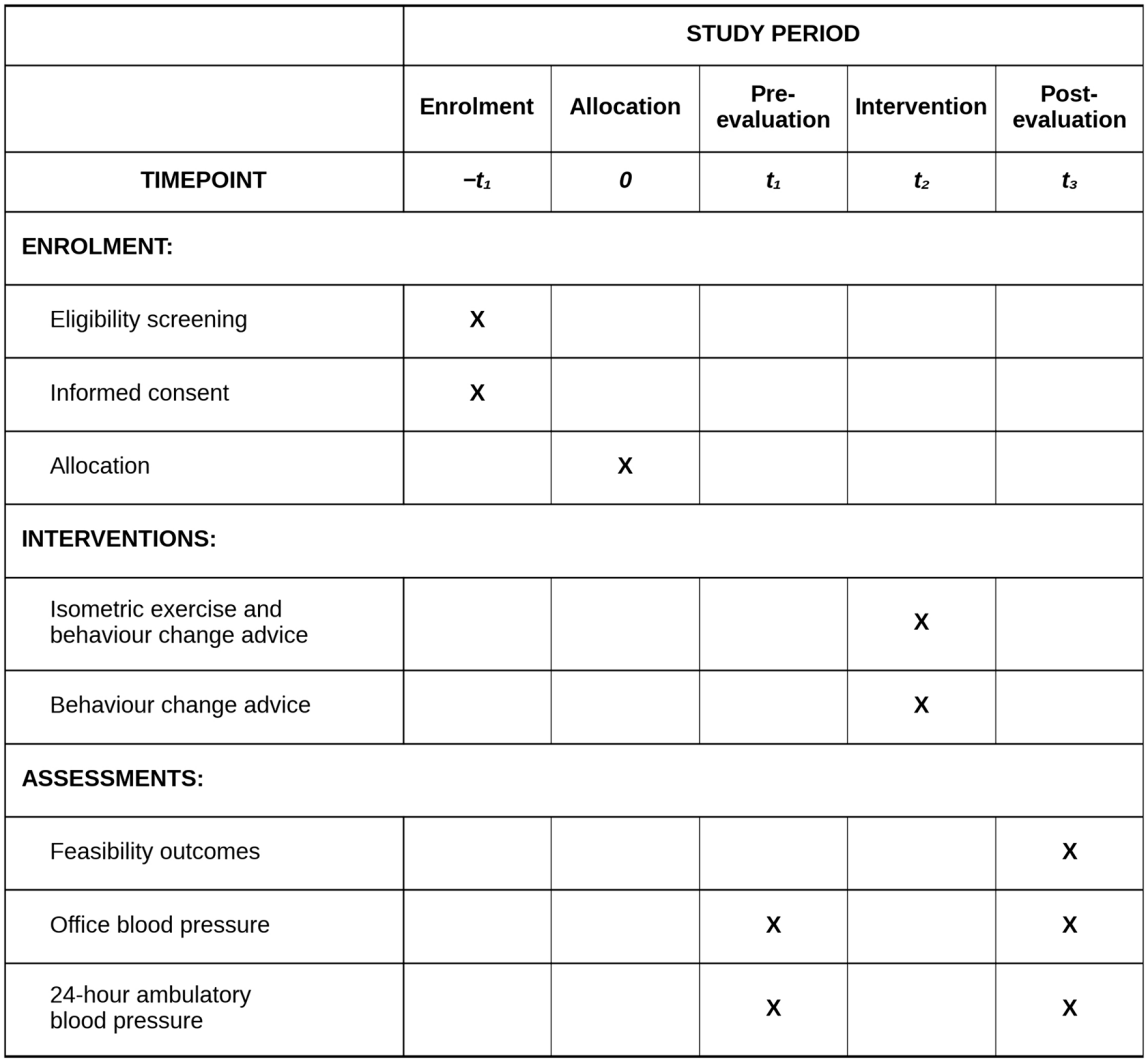
The recommendation of interventional trials (SPIRIT) schedule of enrolment, interventions, and assessments.

This is a single-centred feasibility and pilot randomised controlled trial assessing a remotely supervised leg IET and behavioural change advice for individuals with AH. The Recommendations for Interventional Trials [21] (SPIRIT) flow chart and enrolment schedule, interventions, and assessments for the trial are presented in Fig 1.

### Ethical approval and consent to participate

The study protocol was approved by the Northumbria University Ethics Committee (May 2025, Project ID: 10198), registered, and published in ClinicalTrials.gov (registration number: NCT07213479). The study will be conducted in accordance with the principles of the Declaration of Helsinki. Participants will be provided with appropriate participant information sheets designed in compliance with national guidance. They will have adequate time to consider the information, ask questions and have them answered sufficiently. Participants will be advised that participation is voluntary, and they may withdraw from the study at any time without reason or consequence. Participants who are willing to participate will be asked to sign a consent form, which will be administered by a member of the research team. A copy of the signed consent form will be retained by the investigator and stored in accordance with UK GDPR and the 2018 Data Protection Act, and a further copy will be given to the participant.

### Eligible participants

Participants will be adults (18 years or older) of both sexes recruited through social media and advertisements in the Newcastle upon Tyne area.

### Inclusion criteria

- Diagnosis of AH in accordance with NICE guidelines.
- Under pharmacological treatment for AH with antihypertensive drug, type and dose maintained for the previous four months.
- Blood Pressure with values <180 and <110 mmHg for office systolic and diastolic BP, respectively.
- Not currently engaged in any structured or supervised exercise training programme, including resistance, aerobic, or isometric exercise, defined as planned exercise performed ≥2 times per week at moderate or greater intensity, for at least three months prior to enrolment.

### Exclusion criteria

- Body mass index >35 kg/m^2^.
- Presence of cardiovascular disease beyond AH.
- Known orthopaedic, musculoskeletal, or neurological conditions that restrain isometric exercise execution.
- Presence of secondary AH.
- Inability to follow verbal instructions or complete the study protocol.

### Randomisation

Individuals who meet the study criteria and agree to participate will be randomly assigned to one of two parallel groups: (1) remotely supervised leg IET and behaviour change advice, or (2) a control group behaviour change advice and smartwatch only. Randomisation will be performed with minimisation for sex and the mean of office systolic BP (≤140 mm Hg vs > 140 mm Hg).

### Intervention

The intervention has been co-designed with Northumbria University, Healthworks Newcastle, and patients with AH. The programme consists of:

**Phase 1** (Intensive Phase, 12 weeks) – A structured, high-support period involving thrice weekly online remotely supervised leg IET and behaviour change advice in the format of a lifestyle change leaflet (co-created with patients with AH via patient and public involvement (PPI) meetings to reinforce NICE-recommended standard care [7]. Online resources for each of the behaviour change topics will be created and delivered at weekly timepoints to allow for a steady pace of information delivery. All online resources will remain available to participants throughout the study duration, allowing for multiple reviews and reinforcement of behaviour change.
**Phase 2** (Maintenance Phase, 12–24 weeks) – A transition period focused on sustaining behaviour change with monthly follow-ups and continued guidance.

#### Induction session.

Participants allocated to the intervention will complete an in-person structured induction to ensure familiarity with the technology and intervention. This includes device setup (tablet or mobile phone), online platform training, and guidance on the smartwatch (Huawei Watch D2). Participants will receive an instructional booklet and participate in a test video call to reinforce the skills. Based on our previous study [22], we have included additional support for participants who may encounter challenges with digital literacy, such as difficulties accessing online platforms, internet issues, or syncing the Huawei Watch device. This support offers direct access to our research team for immediate assistance. Based on feedback from our previous PPI meeting, we will also provide tablets and internet connectivity to patients who lack access. This will reduce digital exclusion, support engagement with the intervention, and align with our commitment to equality, diversity, and inclusion.

#### Phase 1 – Intensive programme (12 weeks).

Participants in the intervention group will undergo baseline testing for training intensity prescription by completing an incremental isometric wall squat test (IIWST) [20]. The participant’s squat height position will be ascertained by establishing foot placement, vertical lower limb and trunk positions, and knee joint angles (135°, 125°, 115°, 105°, 95°) (S1 Appendix). Stage 1 of the IIWST will require participants to maintain a squat position of 135° of knee flexion for 2 minutes. Knee joint angle will be decreased by 10° in each subsequent stage until the participant reaches the end of the 95° stage or no longer maintains the knee joint angle within 5° of the target value (volitional fatigue). Once the training knee joint angle has been established, this will be recorded and used to replicate the wall squat position for the remote-based intervention.

#### Supervised exercise programme.

Each session will be delivered online and supervised by exercise professionals or Health Improvement Practitioners, consisting of 4 × 2-minute bouts of wall squat exercise with a 2-minute rest period between bouts.

Squat Position: Participant’s 5-stage wall squat position will be based on individual calculations at baseline. A squat height ruler prototype has been developed to facilitate the home-based individual wall squat height for each stage.

Recording Measurements: After each bout, participants will record: Squat height stage and Borg (CR-10) scale rating (RPE) Target Borg Scale: Bout 1: RPE 4 (target range: 3.5–4.5); Bout 2: RPE 5.5 (target range: 5–6); Bout 3: RPE 7 (target range: 6.5–7.5); Bout 4: RPE 8.5 (target range: 8–9).

Adjustments Based on RPE: If the RPE falls outside the target zone for a given bout, participants will adjust their squat height accordingly (18).

Heart Rate Monitoring: During the session, participants will use the smartwatch (Huawei Watch D2) to continuously monitor their heart rate responses, allowing us to track physiological changes during exercise and ensure participants’ safety.

#### Behaviour change programme.

Behaviour change advice leaflet was built upon established evidence [23,24] adapted from an existing Healthworks intervention and co-produced with public and patient involvement in line with BHF, Blood Pressure UK, and NHS guidelines. The leaflet provides structured lifestyle advice covering: Physical Activity – build towards ≥30 minutes of moderate activity on 5 days/week; step goals are increased by ~10% weekly and monitored via a smartwatch. Weight management: gradual reduction of 5–10% body weight over 3–6 months through sustainable diet and activity changes; Diet: promote ≥5 fruit/vegetable portions daily, reduce salt (<6 g/day), and limit saturated fat, sugar, and processed foods; Alcohol: keep intake <14 units/week with ≥2 alcohol-free days. Caffeine & smoking: reduce caffeine (<4 cups/day); smoking cessation support provided. Mental well-being & sleep: [25], with data uploaded via a QR code or recorded in written format using a BP diary.

#### Phase 2 – Maintenance programme (Months 12–24 weeks).

Following the intensive 12-week intervention, participants will enter a maintenance phase to sustain long-term behaviour change, as studies have shown that extended care in the form of additional contact with treatment providers (typically once or twice a month after the initial treatment) enhances the maintenance of the new lifestyle habit [26]. This phase will be characterised by monthly follow-up phone calls to: Identify barriers to sustaining healthy behaviours; provide ongoing motivation and goal reinforcement; address challenges in maintaining lifestyle changes. During this phase, participants will continue to be encouraged to perform their wall squat training (3 times per week) and to monitor their BP using the Huawei Watch D2 and regularly upload data.

#### Control group.

Participants randomised to the control group will receive the same behaviour change advice as the intervention group, in the format of a lifestyle change leaflet and online resources to reinforce NICE-recommended standard care [7]. They will also receive a smartwatch (Huawei Watch D2) to measure their physical activity and monitor their home BP (similar to the intervention group). Readings will be uploaded via a QR code or recorded in written format using a BP diary. Unlike the intervention group, participants in the control group will not receive direct supervision or ongoing feedback from the research team.

### Outcome measurements

#### Primary outcomes.

**Feasibility outcomes:** Screening, eligibility, recruitment, and retention rates at 12 and 24 weeks.

**Exercise adherence**: Measured as the proportion of prescribed wall-squat sessions completed (from participant logs and device data), as well as mean hold time, squat stage progression, and session frequency.

**Lifestyle adherence:** Assessed by a self-reported questionnaire at 12 and 24 weeks.

**Patient Acceptability:** Determined at 24 weeks through remote, semi-structured 1:1 interviews guided by Sekhon et al.’s Theoretical Framework of Acceptability [27] and the NIH Behavior Change Consortium’s Best Practices and Recommendations [28]. Interviews will continue until data saturation is reached, which is expected to occur with 13–16 participants. Topics covered will explore experience with study participation (informed consent process, time commitment), the acceptability of measures (readability, burden), and experiences, thoughts, and attitudes toward current usual care. All interviews will be audio recorded and transcribed, and these data will be analysed thematically to generate themes and outcomes.

**Focus group discussions:** Clinical exercise physiologists and healthcare clinical professionals will be invited to participate in focus group discussions at regular intervals (~6 months) during the delivery and referral pathway. We will also characterise the remote exercise and behaviour change advice by mapping health behaviour change techniques using the 93-item BCT taxonomy (BCTTv1) [29]. This step will be crucial in informing future work to optimise the intervention and improve the transparency of the intervention content, as well as identify the targeted theoretical mechanisms of action.

#### Secondary outcomes.

This study will assess the feasibility and acceptability of collecting BP data, considering the perspectives of participants and healthcare clinical professionals (Healthworks team), as well as data collection challenges and missing data rates. Findings will inform the suitability of these measures for a future full-scale trial. Assessments will be conducted at baseline (pre-randomisation), 12 weeks, and 24 weeks at Northumbria University, Newcastle upon Tyne, UK. Office BP will be measured using an Omron HEM-742 device. Participants will sit quietly for five minutes, and readings will be taken at one-minute intervals on the right arm until a difference of less than 4 mmHg is achieved between two consecutive measurements. The average measurements will be calculated to determine the office systolic and diastolic BP. Ambulatory BP will be measured using a non-invasive oscillometric device (Welch Allyn ABPM 7100 Central Blood Pressure, USA) worn on the nondominant arm, recording measurements every 15 minutes over 24 hours. Mean systolic, diastolic, and overall BP will be calculated for the full 24-hour period, as well as during awake and asleep periods. BP variability will be assessed using 24-hour standard deviation, weighted SD (SDdn), and average real variability (ARV24).

### Study power

The study will recruit a target sample of n = 70 (intervention n = 35; control n = 35) to estimate feasibility parameters (recruitment, adherence, retention, data completeness, safety) with useful precision and to provide exploratory BP effect sizes (with 95% CIs). As this is a feasibility trial, the study is not powered to test efficacy but to estimate key parameters with acceptable precision. With n = 70, the expected precision is: Retention (assume 75%): Standard error (SE) = √ [0.75 × 0.25 / 70] ≈ 0.052; 95% CI = 75% ± 10% → 65%–85%. Adherence (assume 70%): SE = √ [0.70 × 0.30 / 70] ≈ 0.055; 95% CI = 70% ± 11% → 59%–81% These confidence intervals show that with 70 participants, we can estimate retention and adherence with a margin of error of ~±10%. This is consistent with feasibility study methodology and will provide robust data to inform the design and sample size calculation for a future definitive RCT. Progression to a definitive trial will be guided by predefined traffic light criteria.

### Statistical plan

Feasibility outcomes (screening, eligibility, recruitment, retention at 12 and 24 weeks, and intervention adherence) will be analysed descriptively and presented as counts and percentages with 95% confidence intervals where appropriate.

Baseline characteristics will be summarised by randomised group using appropriate descriptive statistics (mean and standard deviation for normally distributed variables; median and interquartile range for non-normally distributed variables; frequency and percentage for categorical variables). As this is a feasibility study, no formal statistical tests of baseline differences will be conducted.

For BP outcomes (office and ambulatory measures), the number of observed participants at each time point (baseline, 12 weeks, and 24 weeks) will be clearly reported in all outcome tables. In addition, patterns of missing outcome data will be summarised descriptively by group and time point to assess the feasibility of data collection and potential differential attrition. Missingness will be explored using simple descriptive summaries (e.g., counts and percentages by group and visit) to inform planning of a future definitive trial.

Changes in BP over time between intervention and control groups will be explored using linear mixed-effects models, accounting for repeated measures within participants. The primary exploratory model will include fixed effects for group (intervention vs control), time (baseline, 12 weeks, 24 weeks), and the group × time interaction, with a random intercept for participant to account for within-subject correlation.

Given the feasibility nature of the study and sample size (n = 70), modelling will follow a parsimonious approach. The primary model will not routinely adjust for additional covariates. Covariates (e.g., socioeconomic status, medication status, physical activity) will only be included if there is strong justification, such as a clear baseline imbalance or compelling theoretical rationale. Any adjusted analyses will be considered secondary and exploratory.

Model assumptions (e.g., normality of residuals, homoscedasticity) will be assessed graphically. If substantial deviations are detected, appropriate transformations or sensitivity analyses will be considered.

All BP analyses are exploratory and intended to estimate effect sizes with 95% confidence intervals to inform the design and sample size calculation of a future fully powered randomised controlled trial. No formal hypothesis testing for efficacy is planned, and results will be interpreted cautiously in line with feasibility study objectives.

### Trial status

Enrolment of participants will commence in January 2026. The study anticipates a recruitment rate of 4–5 participants per month with recruitment completed by April 2027. The first post-intervention measures (12 weeks) will be performed in April 2026, and the second post-intervention measures (24 weeks) in August 2026. Data collection will finish in October 2027, and statistical analyses completed by February 2028.

## Discussion

To the best of our knowledge, this is the first study to assess the feasibility and acceptability of a remotely delivered, leg IET and lifestyle advice in individuals with AH. This study investigates a novel intervention for managing AH, combining isometric wall squats with support for health behaviour, including physical activity, nutrition, and other cardiovascular risk factors. By offering a remotely supervised program, the intervention is designed to address common barriers regarding exercise for AH, including time, cost, and accessibility.

This research fills a critical gap in the literature, as isometric wall squats have been underexplored in AH populations. Previous studies have primarily focused on normotensive or pre-hypertensive individuals, involved small sample sizes, and had short follow-up. By targeting individuals with AH and incorporating a longer intervention and follow-up period, this study provides a more robust investigation into the potential benefits of this exercise modality. The addition of lifestyle support further distinguishes this work by addressing broader cardiovascular risk factors beyond BP.

This feasibility pilot study was specifically designed to address the common limitations of previous work by recruiting adults (≥18 years) with diagnosed AH from a broad demographic base, reflecting the true clinical and social diversity of this population. Participants will be recruited in partnership with Healthworks Newcastle, a community health charity with over 30 years of experience working in multicultural and socio-economically deprived communities across the North East of England, including members of the Black African, Asian, and Black Caribbean communities.

To improve equity and accessibility, the intervention will be delivered remotely, reducing travel and cost barriers. Participants without digital access will be provided with loaned tablets and mobile data to facilitate participation. All participant materials, including the co-created lifestyle advice leaflet will be available in both hard copy and digital format to ensure accessibility, cultural, and linguistic inclusivity. Recruitment will be continuously monitored by sex, age, ethnicity, and deprivation index, with corrective measures implemented if underrepresentation is identified. Together, these strategies ensure that our study reflects the real-world population most affected by AH, providing an inclusive, scalable, and representative foundation for the future definitive randomised controlled trial.

If successful in demonstrating feasibility, acceptability, and preliminary effectiveness, this study could offer a practical, accessible, and cost-efficient non-pharmacological strategy for AH management, serving as an alternative mode of care that integrates the Voluntary, Community, and Social Enterprise sector with NHS pathways. The findings will provide critical feasibility data to inform the design of a future large-scale, multicentre, randomised controlled trial. Ultimately, this approach could be embedded within existing healthcare structures as a patient-centred model to support long-term AH management and improve overall cardiovascular health.

## Supporting information

S1 Appendix Wall squat protocol. (TIF)

S2 File SPIRIT checklist. (DOCX)
